# Enhanced Activity by Genetic Complementarity: Heterologous Secretion of Clostridial Cellulases by *Bacillus licheniformis* and *Bacillus velezensis*

**DOI:** 10.3390/molecules26185625

**Published:** 2021-09-16

**Authors:** Alexander Arsov, Kaloyan Petrov, Penka Petrova

**Affiliations:** 1 Institute of Microbiology, Bulgarian Academy of Sciences, 1113 Sofia, Bulgaria; alexander_arsov@abv.bg; 2 Institute of Chemical Engineering, Bulgarian Academy of Sciences, 1113 Sofia, Bulgaria; kaloian04@yahoo.com

**Keywords:** cellulose, 2,3-butanediol, *Bacillus licheniformis*, *Bacillus velezensis*, cellulase, heterologous expression, signal peptide, RT-PCR, pBE-S shuttle vector

## Abstract

To adapt to various ecological niches, the members of genus *Bacillus* display a wide spectrum of glycoside hydrolases (GH) responsible for the hydrolysis of cellulose and lignocellulose. Being abundant and renewable, cellulose-containing plant biomass may be applied as a substrate in second-generation biotechnologies for the production of platform chemicals. The present study aims to enhance the natural cellulase activity of two promising 2,3-butanediol (2,3-BD) producers, *Bacillus licheniformis* 24 and *B. velezensis* 5RB, by cloning and heterologous expression of *cel8A* and *cel48S* genes of *Acetivibrio thermocellus*. In *B. licheniformis*, the endocellulase Cel8A (GH8) was cloned to supplement the action of CelA (GH9), while in *B. velezensis*, the cellobiohydrolase Cel48S (GH48) successfully complemented the activity of endo-cellulase EglS (GH5). The expression of the natural and heterologous cellulase genes in both hosts was demonstrated by reverse-transcription PCR. The secretion of clostridial cellulases was additionally enhanced by enzyme fusion to the subtilisin-like signal peptide, reaching a significant increase in the cellulase activity of the cell-free supernatants. The results presented are the first to reveal the possibility of genetic complementation for enhancement of cellulase activity in bacilli, thus opening the prospect for genetic improvement of strains with an important biotechnological application.

## 1. Introduction

Cellulose is the most abundant but least degradable polymer in nature. As the major component of plant biomass and agro-industrial waste, the hydrolyzed form of cellulose is engaged as a substrate in microbial processes for the production of industrial enzymes [1,2], biofuels [3,4], biosurfactants [5], and organic chemicals such as lactic acid, succinic acid, 2,3-butanediol (2,3-BD) [6,7,8,9,10], and many others. However, the natural producers of 2,3-BD do not exhibit cellulase activity sufficient for the direct conversion of cellulose fraction to the target product. This necessitates costly preliminary steps of chemical or enzymatic hydrolysis of cellulose-containing substrates because the decomposition of biomass to soluble sugars is the most important and critical stage in its biochemical conversion into fuels and chemicals [11,12]. The enzymatic saccharification of native or pretreated lignocellulosic biomass to glucose, cellobiose, xylose, arabinose, and mannose need the engagement of lignocellulosic enzyme cocktails, which manifest synergistic cellulolytic, hemicellulolytic, and other lignocellulose-degrading activities [13,14,15].

Recently, several *Bacillus* strains were evaluated as suitable producers of 2,3-BD, since they are generally regarded as safe and may use renewable feedstocks [16,17,18]. Two strains isolated by our team, *Bacillus licheniformis* 24 and *B. velezensis* 5RB, have been pointed out as particularly promising for 2,3-BD production by conversion of plant biomass, as they utilize the sugars in lignocellulose content: glucose, cellobiose, galactose, mannose, xylose, and arabinose, and display some cellulase activity [19].

Investigation of the genetic basis of cellulose and hemicelluloses degradation by bacilli shows that this activity is due to the action of carbohydrate-active enzymes (CAZymes) of the GH families 1, 4, 5, 11, 26, 30, 43, 51, and 53 that include cellulases and xylanases [9,20,21]. The reference genome of *B. licheniformis* contains two gene clusters encoding enzymes for cellulose degradation, rather unlike the closely related *B. subtilis*, which contains none [22]. A recent study of the complete genome of *B. velezensis* NST6, a strain isolated from soil, has not uncovered any special cellulolytic clusters, although it must be noted that 40% of the genes (1588 out of 3924) are with unknown or generally predicted function [23]. Conversely, whole-genome sequencing and annotation of *B. velezensis* 5RB performed by Petrova et al. [24] revealed that the strain has nine genes that may be involved in cellulose hydrolysis. In addition, it owns nine genes related to lignocellulose hydrolysis, and a full cluster for mannan hydrolysis (*gmuB*, followed by *gmuACDREFG*). However, the cellulase activity of *B. licheniformis* 24 and *B. velezensis* 5RB appears to be relatively low and insufficient to obtain fermentable sugars from cellulose.

On the other hand, the most widespread and the most active cellulases are synthesized by other bacteria and fungi [1]. Bacteria of the genera *Clostridium*, *Cellulomonas*, *Thermonospora*, *Ruminococcus*, *Bacteroides*, *Erwinia*, *Thermobifida*, *Streptomyces*, and *Acetivibrio* have been known to be particularly strong producers of cellulases [25]. In our study, we used as genetic source *A. thermocellus*, an anaerobe, spore-forming, thermophilic bacterium of the family *Hungateiclostridiaceae*, which contains one of the most intricate and most efficient cellulolytic systems yet discovered. This so-called cellulosome is a large supramolecular complex of more than 70 different enzymes in addition to a scaffold protein. Leis et al. [26] characterized as cellulases (specifically able to hydrolyze β-1,4-glycosidic bonds) at least 24 enzymes that differed greatly in substrate specificity and hydrolysis products. Cel8A is β-1,4-endoglucanase with a molecular weight of about 50 kDa encoded in the genome of *A. thermocellus*. Together with Cel48S and Cel9K, Cel8A is the most prominent cellulase in the cellulosome, although recent reconstruction studies in vitro show that the importance of these three components is not proportional to their abundance [27]. However, deletion studies suggest that Cel48S, β-1,4-exoglucanase releasing cellobiose residues from the reducing end, is of prime importance. *Cel48S* deletion mutants exhibit a 60% decrease in the cellulose hydrolysis rate, although they retain the ability to solubilize completely 10 g/L crystalline cellulose [28]. Based on structural and functional characteristics of the enzymes, mechanisms of action, and carbohydrate-binding modules peculiarities, the CAZy database classifies endoglucanases in GH5, GH6, GH7, GH8, GH9, GH10, GH12, GH26, GH44, GH45, GH48, GH51, GH74, GH124, and GH148 families, while exoglucanases belong to families GH5, GH6, GH7, GH9, and GH48 [29,30]. Cel8A and Cel48S are members of the GH-M clan that adopts catalytic (α/α)_6_-barrel domain fold [31]. Despite the low sequence similarity, and the different endo/exo mode of action, Cel8A and Cel48S use a common catalytic mechanism to hydrolyze the glycosidic linkage [32]. Cel8A is an inverting endo-β-1,4-glucanase (EC 3.2.1.4), belonging to the GH8 family (formerly known as cellulase family D), since it contains the typical for this family “signature” pattern: a stretch of 19 residues (AT**D**G**D**tlIAwALLrAqkqW), including two conserved aspartates (D). The first of them act as a nucleophile in the catalytic mechanism, while the strictly conserved glutamate (Glu95) residue plays the catalytic role of proton donor [33,34]. In turn, Cel48S is reducing end-acting cellobiohydrolase (EC 3.2.1.176) from the GH48 family (the former cellulase family L). The active site contains residue Glu87 as the general acid catalyst in the cleavage reaction and Asp255 acting as the general base [32]. However, both described cellulases differ greatly from other enzymes with (α/α)_6_-barrel structure, for example, GH126 family, which enzymes lack activity on cellulose and contain other typical conserved sequence regions [35].

The present study aims to enhance the cellulase activity of two potent producers of 2,3-BD—*B. licheniformis* 24 and *B. velezensis* 5RB—by cloning and heterologous expression of *cel8A* and *cel48S* of *A. thermocellus*. The possibility of supplementing bacterial cellulase genes from different families for higher total cellulase activity is discussed.

## 2. Results

### 2.1. Molecular Cloning of Cel8A and Cel48S Genes of A. thermocellus

In order to complement the native cellulase activity of *B. licheniformis* 24 and *B. velezensis* 5RB, two recombinant constructs, containing *cel8A* (1434 bp) and *cel48S* (2226 bp), were developed.

**Figure 1:**
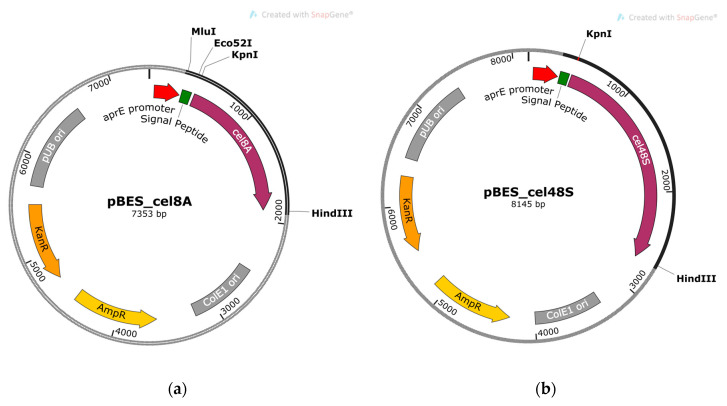
Physical maps of the recombinant constructs containing cellulase genes *cel8A* and *cel48S* of *A. thermocellus* DSM 1237: (**a**) pBES_cel8A; (**b**) pBES_cel48S. The signal peptides library was cloned between *Mlu*I and *Eco*52I sites in pBES_cel8A.

The clostridial genes were obtained as PCR products using chromosome DNA of *A. thermocellus* DSM 1237 as a template and primers ending with *Kpn*I and *Hind*III sites. The PCR fragments were purified, digested with both restriction endonucleases, and cloned in *Kpn*I/*Hind*III linearized shuttle vector pBE-S (5938 bp). The resulting constructs, pBES_cel8A (7353 bp) and pBES_cel48S (8145 bp) shown in Figure 1, were used to tranform *E. coli* strain HST08 and the ampicillin-resistant colonies were collected. The clones containing correctly inserted genes were selected after plasmid extraction, *Kpn*I/*Hind*III restriction digest analysis, and sequencing. Plasmid DNA from the selected *E. coli* clones was isolated on a large scale and used for the transformation of *B. licheniformis* 24 and *B. velezensis* 5RB hosts.

### 2.2. Cellulase Activity of B. licheniformis 24 and Recombinants Containing the Cel8A Gene

**Figure 2:**
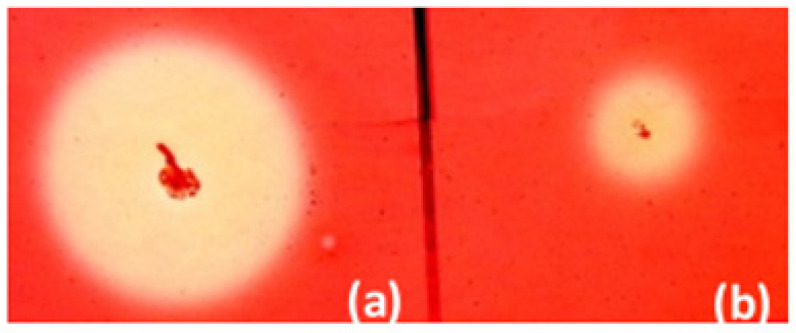
Zones of cellulose hydrolysis formed by the recombinant clone *B. licheniformis* clone 34 containing pBES_cel8A construct (**a**) and *B. licheniformis* host strain (**b**).

Fifty-four kanamycin-resistant and cellulase-positive clones of *B. licheniformis* were selected. They were grown in solid medium supplemented with 0.1% carboxymethyl cellulose (CMC) for 48 h. The largest zone after Congo Red staining was obtained by *B. licheniformis* clone 34 (Figure 2).

**Figure 3:**
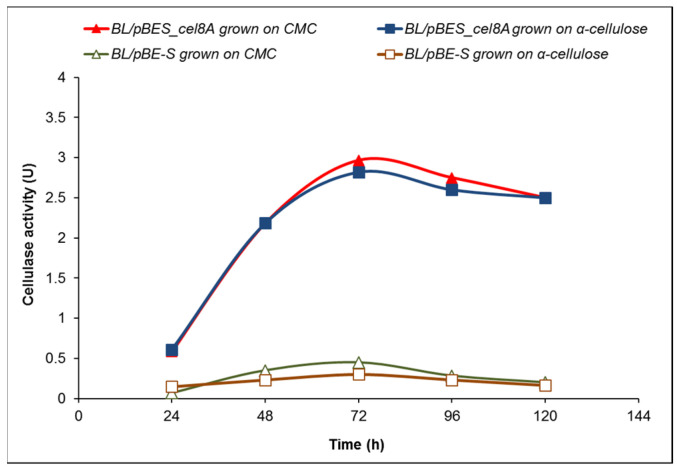
Time course of cellulase activity of *B. licheniformis* 24, containing the expression vector pBE-S (BL/pBE-S), compared to the recombinant clone 34 containing pBES_cel8A construct (BL/pBES_cel8A). The data are mean values from three batch processes.

The cellulase activity of *B. licheniformis* 24 clones containing the expression vector pBE-S and pBES_cel8A construct (clone 34) was compared in a course of batch processes in a fermentation medium containing either 10 g/L CMC or 10 g/L α-cellulose (Figure 3).

The highest activity of clone 34 (BL/pBES_cel8A) grown in the presence of CMC reached 2.97 U at 72 h, while the highest value for the host containing the expression vector (BL/pBE-S) was approximately 0.4 U. When grown on 10 g/L crystalline α-cellulose, the enzyme activity of clone 34 followed similar kinetics to that in medium with CMC, with slightly lower values, while the cellulase activity of the control remained between 0.2 and 0.3 U.

**Figure 4:**
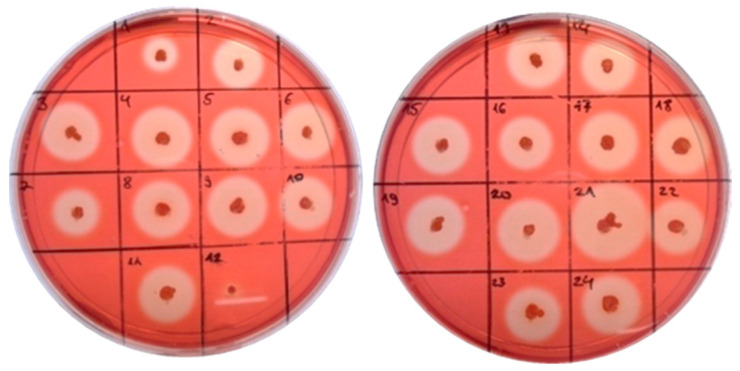
Zones of cellulose hydrolysis formed by the recombinant clones of *B. licheniformis* containing pBES_cel8A construct with signal peptides library.

The further enhancement of the cellulase activity continued via the cloning of the sequences encoding different signal peptides (SP) upstream of the gene *cel8A*. We used In-Fusion^®^ cloning with the recombinant pBES_cel8A construct and selected eight clones with different signal peptides, five with the largest zones (5, 6, 20, 21, 34) and three (1, 19, 23) with smaller (Figure 4). All eight signal peptides were confirmed by sequencing and further analyzed to determine their protein sequence and theoretical probability (Table 1).

**Figure 5:**
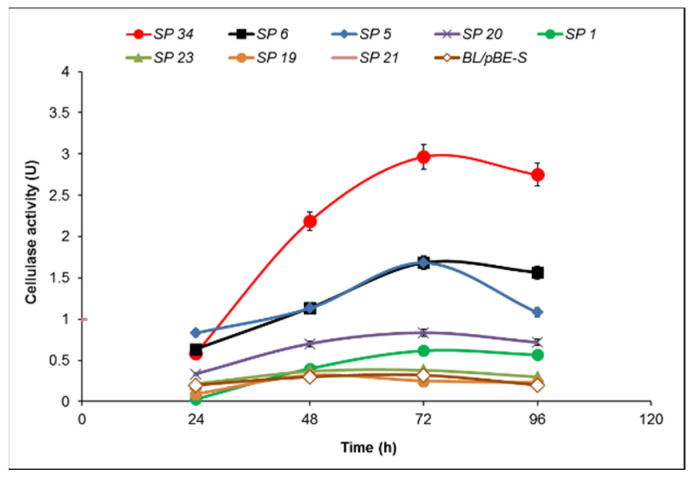
Time course of cellulase activity of *B. licheniformis* recombinant clones containing the pBES_cel8A construct with a library of signal peptides compared to *B. lichenifromis* 24 transformed with pBE-S vector (control). The data are mean values from two batch processes.

A strict correlation between the size of the hydrolysis zone and the cellulase activity was not observed. The subtilisin-like signal peptide with one amino acid substitution (methionine to valine) as compared to subtilisin SP did show the highest activity—2.97 U at 72 h, more than eight times higher than the host strain containing pBE-S vector at the same time point (Figure 5).

Six of the signal peptides produced activities not wholly consistent with their hydrolysis zones. Signal peptides SP 5 and SP 6 exhibited the highest activity (in agreement with their relatively large zones) at 72 h of cultivation, reaching activity 1.68 U, almost six times more than that of the control (BL/pBE-S) at the same time point. Signal peptide 20, albeit comparable in the zone, lagged in its activity; it did peak at 72 h but managed less than three times higher activity compared to the control. Most surprising was SP 21, with one of the largest hydrolysis zones, yet the lowest enzyme activities of all. In contrast, the zone formed by SP 1 was comparable to that of the host strain, but its enzyme activity was three times higher at 96 h (0.6 U vs. 0.23 U). The other two signal peptides (19 and 23) showed equal or lower activity at the different time points, as shown in Figure 5.

### 2.3. Cellulase Activity of B. velezensis 5RB Recombinants Containing the Cel48S Gene

**Figure 6:**
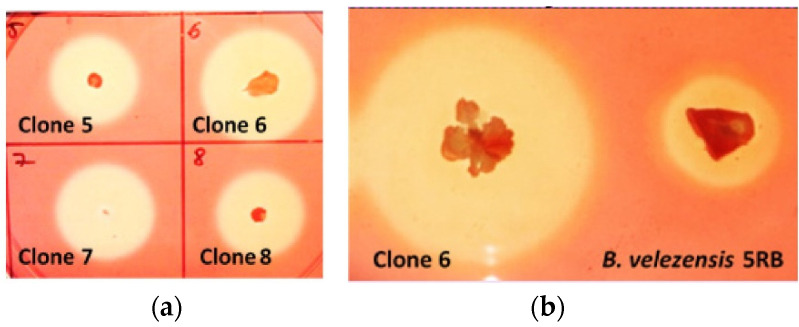
Zones of cellulose hydrolysis formed by recombinant *B. velezensis* clones containing the pBES_cel48S construct (**a**) and the selected clone 6 in comparison with the host strain (**b**).

*B. velezensis* 5RB was transformed with the construct pBES_cel48S (Figure 1b), containing the clostridial *cel48S* coupled with the sequence encoding the subtilisin-like signal peptide. Clone selection of *B. velezensis* yielded several recombinants with large and promising hydrolysis zones. Clone 6 was selected for further analysis based on the best ratio between the size of the colony and the size of the zone (Figure 6).

**Figure 7:**
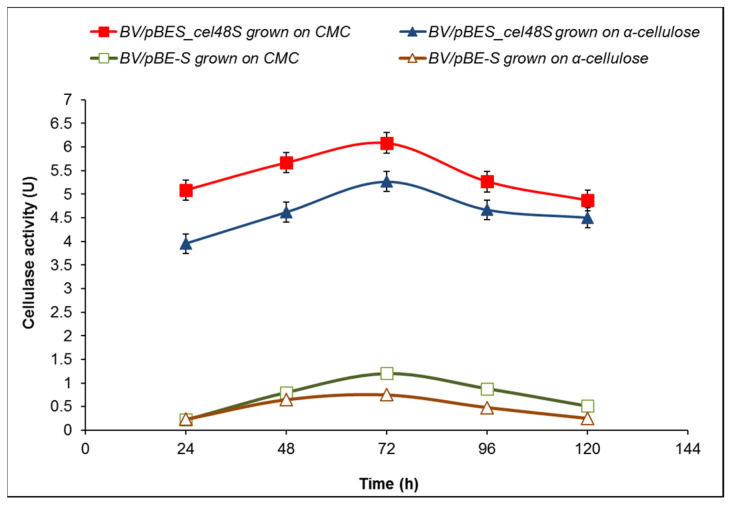
Time course of cellulase activity of *B. velezensis* recombinants bearing the expression vector pBE-S or the construct pBES_cel48S (clone 6). The fermentation media contained 10 g/L CMC or 10 g/L α-cellulose. The data presented are mean values of three batch processes.

The cellulase activity of the selected *B. velezensis* clone 6 grown in the presence of 10 g/L CMC peaked at 72 h reaching 6.08 U, more than five times higher than that of the control BV/pBE-S (*B. velezensis* 5RB containing the expression vector pBE-S). Interestingly, the exchange of CMC with α-cellulose as a carbon source led to the even higher activity of clone 6 compared to the control (5.27 vs. 0.75 U at 72 h), which represents a seven-fold increase in total cellulase activity (Figure 7).

### 2.4. Cellulase Genes Transcription Profiles

To confirm the expression of the cloned clostridial genes in the recombinant clones and to elucidate the expression of the native cellulases of both hosts on the transcription level, the method of reverse-transcription PCR (RT-PCR) was used. NCBI and KEGG databases mining revealed that *B. licheniformis* reference genome contains three genes for cellulases: CelA (654 amino acids, GH 9), CelB (560 amino acids, GH5), and CelC (514 aa, GH5). Thus, the complete genome sequence NC_006270 of *B. licheniformis* ATCC 14580 was used for primer design. For *B. velezensis* 5RB was traced the sequence of the main acting cellulase gene, *eglS*. It was obtained from the whole genome sequencing (WGS) of the strain, with accession no. QXJL01000001 [24]. Primer pairs, specific to each cellulase gene and the expected RT-PCR products, are listed in Table 2.

**Figure 8:**
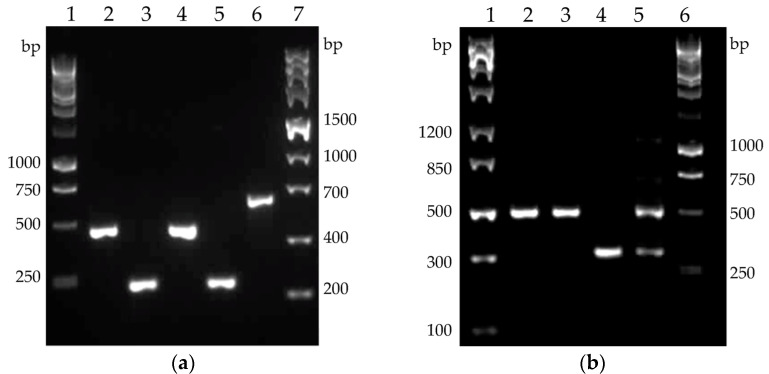
Reverse-transcription PCR revealing the cellulase genes expression. (**a**) Cellulase genes expressed in *B. licheniformis* host and recombinant clone 34. Lanes and samples: (1) Perfect Plus™ 1 kb DNA Ladder; (2) *B. licheniformis* host *celB*; (3) *B. licheniformis* host *celA*; (4) *B. licheniformis* clone 34 *celB*; (5) *B. licheniformis* clone 34 *celA*; (6) *B. licheniformis* clone 34 *cel8A*; (7) ZipRuler Express DNA Ladder 2; (**b**) Cellulase genes expressed in *B. velezensis* host and recombinant clone 6. Lanes and samples: (1) ZipRuler Express DNA Ladder 1; (2) *B. velezensis* host *eglS*; (3) *B. velezensis* clone 6 *eglS*; (4) *B. velezensis* clone 6 *cel48S*; (5) *B. velezensis* clone 6 *eglS* and *cel48S*; (6) Perfect Plus™ 1 kb DNA Ladder. The electrophoretic patterns were obtained by the use of 1.5% agarose gel, run at 60 V for 75 min.

The two *Bacillus* species differ markedly in their native cellulase profile (Figure 8). Of the three cellulase genes in *B. licheniformis*, *celA* and *celB* were found to be expressed on mRNA level in both the host strain and the recombinant clone. The expression of the gene *celC* encoding putative third cellulase (514 amino acids) was not observed in any samples (data not shown). The PCR product of clostridial cellulase *cel8A* also perfectly matched the expected size. The single native cellulase of *B. velezensis*, encoded by the gene *eglS*, was found to be expressed on mRNA level in both the host strain and the recombinant clone 6, which also contained mRNA of the clostridial cellulase *cel48S* (Figure 8b).

## 3. Discussion

Bacterial cellulases are divided into three major types: endoglucanases (EC 3.2.1.4), cellobiohydrolases (EC 3.2.1.91), and β-glucosidases (EC 3.2.1.21) [36]. In nature, the soil-derived cellulose-degrading microbial community usually secretes mixed enzymes that act synergically. Metagenome studies including reconstruction of bacterial draft genomes are applied to elucidate the full spectrum of acting enzymes in a certain ecological niche [37]. Saprophytic soil and rhizosphere bacilli of *B. subtilis* complex, which includes *B. licheniformis* and *B. velesensis*, are an important part of these cellulose-converting bacterial clusters. *B. licheniformis* has recently aroused biotechnological interest in the production of bioactive compounds for aquaculture and agriculture, food, biomedicine and pharmaceutical industry, bioflocculation, biomineralization, biofuel production, and bioremediation. *B. velezensis* is known as PGPR (plant growth-promoting rhizobacterium), first isolated in 2005, and belonging to *B. amyloliquefaciens* “operational group” [38]. The growing interest in *B. velezensis* is inspired by its ability to produce substances promoting plant growth or protecting plants from various pathogens [39,40]. In addition, both species are valuable producers of platform chemicals and have been studied for inclusion in numerous industrial processes [22]. *B. licheniformis* strain 24, used as a host in this study, is among the best nonpathogenic producers of 2,3-BD from glucose [41]. *B. velezensis* 5RB has an even higher potential to produce 2,3-BD from biomass, since it converts all sugars in lignocellulose composition, but also inulin and starch [19]. However, the application of both strains in simultaneous saccharification and fermentation (SSF) processes of plant biomass conversion is hindered by their limited cellulase activity. In order to enhance the natural cellulase activity of *B. licheniformis* and *B. velezensis*, the genes encoding two of the most important components of the clostridial cellulosome: *cel8A*, encoding endo-1,4-β-d-glucanase, and *cel48* for exo-1,4-β-d-glucanase were introduced in these hosts. Clostridial cellulosome comprises a wide variety of polysaccharide degrading enzymes (e.g., cellulases, hemicellulases, and pectinases), and scaffold proteins. To clarify the importance of each component, Hirano et al. [27] reconstituted the complex of cohesin protein CipA, and the three major cellulosomal cellulases Cel48S, Cel8A, and Cel9K at a molar ratio 4.06:1.82:0.72. However, towards crystalline cellulose, the complex was less active in comparison to the native cellulosome. The same team reconstituted the cellulosome complex from 3, 12, 30, and 40 components using a wheat germ cell-free protein synthesis system and showed that fewer than nine enzymes are crucial for cellulose degradation, but some noncellulosomal cellulases, such as Cel9I and Cel48Y also play a role in the activity for crystalline cellulose [27]. Cel48S accounts for ~30% of the weight of the cellulosome isolated from the Avicel-grown culture. Cel48A weight level decreased to ~10% when the culture was grown on cellobiose, implying that this enzyme is crucial for crystalline cellulose hydrolysis [42].

To date, an extracellular production of cellulases has been reported for several strains of *B. licheniformis*: KIBGE-IB2, NCIM 5556, YNP5-TSU, and BCLLNF-01 [37,43,44,45]. However, the genetic basis of this activity has not been discussed in these works. In our study, the expression of *celA* and *celB* of *B. licheniformis* 24 was confirmed by RT-PCR (Figure 8a). There was no specific PCR product obtained for *celC* (data not shown), suggesting that *celC* is rather a pseudogene. These results confirm the findings of de Araújo et al. [46], who isolated and characterized the enzyme CelA of *B. licheniformis* ATCC 14580 (named by the authors BlCel9). Kim and Ku [47] reported the successful over-expression of *celA* in *E. coli* and the display of the cellulase on the cell surface by fusion of the enzyme to an outer membrane-bound ice-nucleation protein of *Pseudomonas syringae*. CelA (BlCel9) shows similarities to the other GH9 cellobiohydrolases of *A. thermocellus*, for instance, CbhA [48]. In our study, for complementation of the natural cellobiohydrolase activity of CelA of *B. licheniformis* 24, Cel8A was used—an enzyme with endoglucanase activity, from the GH8 family, and known for its high activity and stability. Although natural cellulase activity of wild-type *B. licheniformis* is retained intracellularly (22.7% in the periplasm, and 61.3% in the cytoplasm) [47]; in our study, by the overexpression of *cel8A* gene was reached more than an eight-fold enhancement of extracellular cellulase activity (in the cell-free supernatant). This was achieved by the successful fusion of *cel8A* to a sequence encoding signal peptides library, especially designed for *B. subtilis*. Thus, a signal peptide, almost identical to that of subtilisin (with one amino acid substitution—methionine to valine) was found to be the most effective leader that could transport the heterologous protein through the cell membrane.

Concerning *B. velezensis*, a natural cellulase activity of this species was described for the strains ZY-1-1 [49], ASN1 [50], and FAY0103 [51]. The complete genome of that used as a host *B. velezensis* 5RB revealed the presence of *eglS* gene encoding endo-1,4-β-d-glucanase (EC 3.2.1.4) [24]. In our work, these in silico data were confirmed on transcriptional level by RT-PCR, which showed *eglS* expression both in the host and recombinant *B. velezensis* 5RB (Figure 8b). Although several genes related to cellulose degradation presented in the draft genome, such as *bglA*, *bglC*, *bdlH*, *licH*, and *gmuD*, encoding 6-phospho-β-glucosidases (GH4); *lacG* for 6-phospho-β-galactosidase (GH1), and *bglS* for β-glucanase (GH16), there is a lack of genes encoding cellobiohydrolases in *B. velezensis*. That is why, to enhance the cellulase activity, the clostridial *cel48S* gene of the GH48 family was chosen to be over-expressed in *B. velesensis* 5RB. This genetic complementation resulted in the secretion of Cel48S reaching the highest reported value of extracellular cellulase activity in this species. The predominant presence of the enzyme in the cell-free supernatant may be explained by its charge. Investigating the influence of the charge of secretory proteins in *B. subtilis*, Stephenson et al. [52] observed that proteins with isoelectric point pI lower than 7.0 are found predominantly in the culture medium, while those with pI higher than 7.0 remain attached to the cell surface. Notably, according to the ExPASy pI computation, enzyme Cel48S has pI = 5.26, thus having an appropriate electrostatic charge to be successfully secreted. Moreover, the enzyme Cel48S displayed the expected high affinity to crystalline cellulose (Figure 7), similar to its activity in clostridial cellulosome [27,42,53].

The achievements of the present work consist in heterologous expression and the successful extracellular secretion of two different cellulases originating from a highly cellulolytic strain *A. thermocelus* in *B. licheniformis* and *B. velesensis*. Endocellulase Cel8A (GH8) facilitated the action of the major *B. licheniformis* exo-cellulase CelA (GH9). Conversely, in *B. velezensis*, cellobiohydrolase Cel48S (GH48) successfully complements the action of endocellulase EglS (GH5). However, despite the significant increase in the total cellulase activity of the strains, it turns out that all recombinants remain unable to perform direct hydrolysis of cellulose substrates to fermenting sugars and to produce 2,3-BD. One reason is the insufficient cellulase activity, probably due to the thermophilic or the dockerin-bearing nature of the cloned enzymes. Another reason is that *Bacillus* cellulases display the best activity against somewhat unusual substrates. For example, de Araújo et al. [46] reported that the natural cellulase BlCel9 of *B. licheniformis* has the best action against substrate PASC—the phosphoric acid swollen cellulose, followed by bacterial cellulose, which fibers are very small in size (0.001 μm), filter paper, and to a lesser extent, CMC. In addition, sugars produced by CelA (BlCel9) are cellotetraose and cellotriose, although theoretically the predominant products should be cellobiose and glucose.

Another problem of the potential SSF process for 2,3-BD production from cellulose is the need to use media containing microelements. However, some (Cu, Fe, Co) are inhibitors of enzymes in vivo, in contrast to the in vitro assay, where the enzyme activity is tested in a buffer containing pure substrate, without the presence of metal ions.

For the direct synthesis of 2,3-BD directly from cellulose, the total cellulase enzyme activity of microbial 2,3-BD producers must be further increased by careful analysis of the composition of the medium and the cultivation conditions. In addition, more appropriate plant substrates should be selected, i.e., in addition to the hard-to-degrade cellulose; they have to contain mannans, galactans and xylans, which would allow the engagement of the broad glycoside-hydrolase enzyme profile of the bacilli. At present, however, the task of eliminating the stages of pre-hydrolysis of cellulose and performing SSF processes by *Bacillus* cellulase producers seems difficult to perform.

## 4. Materials and Methods

### 4.1. Bacterial Strains, Media and Cultivation Conditions

*B. licheniformis* strain 24 and *B. velezensis* 5RB were isolated from soil and lake sediment in Bulgaria [19,24] and is stored in the microbial culture collection of the Institute of Microbiology, Bulgarian Academy of Sciences.

*B. licheniformis* 24 was identified by 16S rDNA sequencing (NCBI GenBank accession no. MK461938); *B. velezensis* 5RB was affiliated to this species after WGS, accession no. QXJL01000001.

*Escherichia coli* HST08 strain (STELLAR^TM^ competent cells, genotype F-, endA1, supE44, thi-1, recA1, relA1, gyrA96, phoA, Φ80d lacZΔ M15, Δ(lacZYA-argF) U169, Δ(mrr-hsdRMS-mcrBC), ΔmcrA, λ-) was purchased from Clontech Laboratories, Inc., A Takara Bio Company (Mountain View, CA, USA).

*E. coli* was grown in Luria–Bertani (LB), or SOC medium, supplemented with 15 g/L agar when needed. *B. licheniformis* 24 and *B. velezensis* 5RB were grown in the medium described by Okonkwo et al. [54], modified by Petrova et al. [19], with the following content (g/L): glucose, 20, or CMC, 10; yeast extract, 5; tryptone, 5; (NH_4_)_2_SO_4_, 3; KH_2_PO_4_, 3.5; K_2_HPO_4_, 2.75; MgSO_4_, 0.2; ammonium acetate, 1.5; CoCl_2_ 6H_2_O, 0.09; salt solution, 3 mL per liter, containing (g/L): FeSO_4_, 0.4; H_3_BO_3_, 0.8; CuSO_4_·5H_2_O, 0.04; NaMoO_4_·2H_2_O, 0.04; MnCl_2_·4H_2_O, 5.0; ZnSO_4_·7H_2_O, 0.1; Co(NO_3_)_2_·6H_2_O, 0.08; CaCl_2_·2H_2_O, 1.0; Biotin, 0.01.

Bacterial cultures were incubated in Erlenmeyer flasks of 500 mL containing 100 mL medium and 10% inoculums, which were prepared by overnight incubation of single colonies in 50 mL fermentation medium.

Fermentations of *Bacillus* spp. for cellulase enzyme activity determination were performed at 37 °C, and 140 rpm in GFL 1092 rotary shaker (GFL Gesellschaft für Labortechnik mbH, Burwedel, Germany). Samples were taken at 12, 24, 48, and 72 h. Supernatants were separated by centrifugation at 12,000× *g* for 10 min on a Model 1–14 centrifuge (Sigma, Osterode am Harz, Germany).

### 4.2. Molecular Cloning of Cel8A and Cel48S

The genes *cel8A* and *cel48S* were amplified with a template total DNA of *A. thermocellus* DSM 1237 (=ATCC 27405) purchased from the Leibniz Institute (Braunschweig, Germany).

PCR amplification was performed in QB-96 Satellite Gradient Thermal Cycler (LKB Vertriebs GmbH, Vienna, Austria) with primers specially designed to contain *Kpn*I and *Hind*III restriction sites. The sequences of the used primers are presented in Table 3.

The optimal annealing temperature was determined to be 67.9 °C. PCR reactions consisted of 15 ng DNA template, 0.4 µM primers, Premix Ex Taq Hot Start Version (Clontech Laboratories, Inc., A Takara Bio Company (Mountain View, CA, USA)), and sterile water to 25 µL final volume. Between initial denaturation for 3 min and 30 s on 98 °C and final elongation for 5 min at 72 °C, the following temperature profile was used for 38 cycles: 10 s denaturation at 98 °C, 45 s annealing at 67.9 °C, and 2.5 min elongation at 72 °C.

The PCR fragments (1434 bp for *cel8A* and 2226 bp for *cel48S*) thus obtained, after restriction with *Kpn*I and *Hind*III, were cloned into pBE-S shuttle vector, purchased from Clontech Laboratories, Inc., A Takara Bio Company (Mountain View, CA, USA), under the subtilisin (*aprE*) promoter and signal peptide. The recombinant construct was transformed in *E. coli* STELLAR^TM^ competent cells.

The proper cloning was confirmed by sequencing (Macrogen Inc., Amsterdam, The Netherlands). Plasmid DNA from *E. coli* clones was purified with Plasmid Miniprep DNA Purification Kit (EURx^®^, Gdańsk, Poland) to obtain a sufficient amount of plasmid DNA for linearization with *Mlu*I and *Eag*I and subsequent In-Fusion^®^ cloning (Clontech Laboratories, Inc., Mountain View, CA, USA) with a library of 173 signal peptides.

The cloning of different signal peptides was confirmed by sequencing with a custom-designed primer by Macrogen Inc. (Amsterdam, The Netherlands) (Table 3).

DNA fragments were visualized using gel electrophoresis on 1–1.5% agarose (AlfaAesar, Kandel, Germany), in TAE buffer (40 mM Tris-base, 20 mM acetic acid, 1 mM EDTA), and stained with SYBR Green I (Thermo Scientific Inc., Waltham, MA, USA).

### 4.3. Transformation and Selection of the Recombinant Clones

*E. coli* transformation was carried out following Protocol PT5055-2 of STELLAR^TM^ competent cells manufacturer’s instructions.

Transformation of *B. licheniformis* 24 and *B. velezensis* 5RB with the recombinant constructs or the plasmid library was performed via electroporation following a modified version of the high-osmolarity protocol by Xue et al. [55]. Briefly, overnight culture in standard LB media (1% tryptone, 0.5% yeast extract, 0.5% NaCl) was diluted 16 times in LB media with 0.5 M sorbitol in 500 mL Erlenmeyer flask and grown for 2 to 3 h until OD600 of 0.9 was reached. The flask was chilled on ice for 10 min and the bacteria were washed four times with an ice-cold electroporation medium (0.5 M sorbitol, 0.5 M mannitol, 10% glycerol). After the final centrifugation (3350 g/10 min/4 °C), the competent cells were resuspended in 625 µL electroporation medium. Aliquots of 60 µL were used for electroporation in ice-cold GenePulser cuvettes with 0.1 cm electrode gap on MicroPulser electroporator (BioRad Laboratories, Hercules, CA, USA). Pulse of 2.1 kV was applied for 4–5 ms and 1 mL recovery medium (0.5 M sorbitol and 0.38 M mannitol in LB) was added as quickly as possible. The culture was transferred into 15 mL glass tubes, incubated for 3 h, then spread on Petri dishes with LB-agar and left overnight at 37 °C. Competent cells were stored at −70 °C and reused several times with a minimal loss of electroporation efficiency.

The selection of *E. coli* transformants was carried out using solidified LB medium, containing 50 µg/mL ampicillin. For the selection of *Bacillus* spp. clones, kanamycin with a final concentration of 5 µg/mL (liquid media) or 10 µg/mL (agar media) was added.

### 4.4. RT-PCR for Cel8A, Cel48S and Native Bacillus spp. Cellulases

Total RNA was isolated with the GeneMATRIX Universal RNA Purification Kit (EURx^®^, Gdańsk, Poland); 300 ng was treated with DNase I for 30 min at 37 °C, followed by enzyme inactivation for 10 min at 65 °C in the presence of 20 mM EDTA. The reverse transcription was done with NG dART RT kit using random hexamer primers (EURx^®^, Gdańsk, Poland). RNA and cDNA concentrations were measured on a Quawell UV Spectrophotometer Q3000 (Quawell Technology Inc., San Jose, CA, USA). For RT-PCR, Premix Ex Taq Hot Start Version (Clontech Laboratories Inc., Mountain View, CA, USA) was used together with the following temperature profile: 10 s denaturation at 98 °C, 30 s annealing at 55 °C, and 1 min and 10 s elongation at 72 °C.

### 4.5. Cellulase Activity Assay

Cellulase activity in supernatants was measured with the DNS method of Miller [56]. The supernatants were diluted five times in phospho-citrate buffer (0.16 M Na_2_HPO_4_, 0.02 M citrate, pH 7) with 1% CMC as substrate and then incubated for 1 h at 50 °C. The reaction was stopped by adding an equal volume of DNS. The OD_540_ of each sample was measured against its control using a Helios Omega UV-VIS spectrophotometer (Thermo Scientific, Waltham, MA, USA). The amount of glucose released was estimated with a standard curve of concentrations between 1 and 0.0625 µmol/mL. One unit of cellulase activity is defined as the enzyme that releases 1 μM glucose for 1 min. Cellulase activity of the selected clones was also estimated on agar medium with 0.1% CMC by the size of the hydrolysis zone. The plates were stained with 0.1% Congo Red solution (Merck KGaA, Darmstadt, Germany) for 15 min and de-stained with 1 M NaCl for as long as necessary to obtain bright zones.

### 4.6. Analytical Methods

The fermentation products (cellobiose, glucose, 2,3-BD, acetoin, ethanol, and organic acids) were analyzed using YL Instrument 9300 HPLC System (YL Instrument Co., Ltd., Anyang, Korea), by HPLC column Aminex HPX-87H (BioRad Laboratories, CA, USA) at 65 °C. The mobile phase was 5 mmoL H_2_SO_4_ at a flow rate of 0.6 mL/min. All compounds were detected by the RI detector (YL 9170 RI Detector). All standard substances were purchased from Merck KgaA, Darmstadt, Germany.

### 4.7. Bioinformatics Tools

The nucleotide sequences were analyzed by the program ChromasPro 2.1.10 (https://technelysium.com.au/wp/, accessed on 23 August 2021). The plasmid constructs maps were done with SnapGene (GSL Biotech LLC, Chicago, IL, USA). Other resource portals used for bioinformatics analysis were BLAST (NCBI), ClustaW, Expasy (Swiss Institute of Bioinformatics), and KEGG database (Kyoto, Japan). SignalP 5.0 software of DTU Bioinformatics at Technical University of Denmark (http://www.cbs.dtu.dk/services/SignalP/, accessed on 23 August 2021) was used for the prediction of signal peptides [57].

## 5. Conclusions

The present work is the first to reveal that the approach of genetic complementation of cellulase functions increases the total cellulase activity in bacilli with industrial application. For the first time, genes for clostridial cellulases have been successfully expressed and secreted in *B. licheniformis* and *B. velesensis*. The expression of natural cellulase genes in strains of both species was demonstrated by transcriptome analysis. The heterologous expression of *cel48S* enhanced the cellulolytic capability of *B. velezensis* by more than twice, whereas that of *cel8A* increased the cellulase activity of *B. licheniformis* over ten-fold. The selection of signal peptides showed that subtilisin-like SP is the most effective in protein transport across the cell membrane of bacilli. Thus, the successful expression and secretion of clostridial cellulases open a new prospect for future improvement of *Bacillus* strains.

## Figures and Tables

**Table 1:** Signal peptides sequences and their characteristics.

Clone	Signal Peptide Sequence *	Sec/SPI Likelihood	AA	Cleavage Site
34	VRSKKLWISLLFALTLIFTMAFSNMS*AQA*	0.916	29	29–30
6	MFKKLLLATSALTFSLSLVLPLDGH*AKA*	0.932	28	28–29
5	MKKTIMSFVAVAALSTTAFG*AHA*	0.960	23	23–24
21	MKKKKTWKRFLHFSSAALAAGLIFTSAAP*AEA*	0.933	32	32–33
23	MKRLFMKASLVLFAVVFVFAVKGAP*AKA*	0.906	28	30–31
20	MKKRLIGFLVLVPALIMSGITL*IEA*	0.863	25	27–28
1	MKVCQKSIVRFLVSLIIGTFVISVPFM*ANA*	0.858	30	30–31
19	MKRLLSTLLIGIMLLTCAPS*AFA*	0.850	23	23–24

* Hydrophobic amino acids are underlined. The SP cleavage site is in italics.

**Table 2:** The detected cellulase genes, specific primer pairs for its determination, and the size of RT-PCR expected products.

Target	Gene	Primer Pair ^1^	Product Size (bp)
*B. licheniformis* 24	*celB* (GH5)	BLcelB_F, BLcelB_R	451
*B. licheniformis* 24	*celA* (GH9)	BLcelA_F, BLcelA_R	232
*B. licheniformis* 24	*celC* (GH5)	BLcelC_F, BLcelC_R	407
*B. velezensis* 5RB	*eglS* (GH5)	eglS_F, eglS_R	502
Clone 34	*cel8A* (GH8)	RTCel8AF, RTCel8AR	614
Clone 6	*cel48S* (GH48)	RTCel48SF, RTCel48SR	327

^1^ The sequences of the primers are listed in Table 3.

**Table 3:** Primers used in this study.

Primer	Sequence (5′–3′) ^1^	Target Sequence	Positions in Gene	Tm (°C)	Purpose
Cel8A_F	CAGTCGGTACCGTGAAGAACGTAAAAAAAAGAGTAGGTGTGG	CP000568	1–31	77.9	Cloning
Cel8A_R	CTAGTAAGCTTCTAATAAGGTAGGTGGGGTATGCTCTTTATC	CP000568	1403–1434	76	Cloning
Cel48S_F	CAGTCGGTACCATGGTAAAAAGCAGAAAGATTTCTATTCTG	CP000568	1–30	74.9	Cloning
Cel48S_R	CTAGTAAGCTTTTAGTTCTTGTACGGCAATGTATCTATTTC	CP000568	2196–2226	72.9	Cloning
SP_F	CTTAAGCAAAAGGAGAGGGACGCGT	pBES_cel8A	312–337	67.4	Sequencing
SP_R	GTAGGTGTGGTTTTGCTGATTCTTGC	pBES_cel8A	475–502	64	Sequencing
RTCel8AF	TCTTGCAGTGTTGGGGGTTT	CP000568	42–61	58.4	RT-PCR
RTCel8AR	CAGGCGCAAAATATGACGGG	CP000568	655–636	60.5	RT-PCR
RTCel48SF	CTGCATTCGCAGGTCCTACA	CP000568	71–90	60.5	RT-PCR
RTCel48SR	AAGAAGACACATCCGGCTGC	CP000568	397–378	60.5	RT-PCR
eglS_F	AACAAAGATTCAACGAAGGACGCC	NZ_CP011937	994–1017	63.5	RT-PCR
eglS_R	TGGGTTCTGTTCCCCAAATCA	NZ_CP011937	1495–1475	59.4	RT-PCR
BLcelB_F	GCCAATGAAAAGCATTCAAAATGG	WP_011197981	901–924	60.1	RT-PCR
BLcelB_R	CCATAACCTGGCTGGCTCC	WP_011197981	1351–1333	61.7	RT-PCR
BLcelC_F	GGTTCGTCAGGAGACTCTGG	CP034569	1000–1019	62.5	RT-PCR
BLcelC_R	ATCTGGATTTCGCCAGTATCAG	CP034569	1406–1385	60.3	RT-PCR
BLcelA_F	CGATTTAACGGGAGGGTGGT	LR134392	252–271	60.5	RT-PCR
BLcelA_R	GTCTAGAGCGCCATTTCCGA	LR134392	483–464	60.5	RT-PCR

^1^ The underlined sequences represent the sites recognized by endonucleases: *Kpn*I (Cel8A_F, Cel48S_F) and *Hind*III (Cel8A_R, Cel48S_R).

## Data Availability

Not applicable.

## Sample Availability

The host strains *B. licheniformis* 24 and *B. velezensis* 5RB are available from the authors.
